# Supplementary figures and images for: Melatonin Prevents Chondrocyte Matrix Degradation in Rats with Experimentally Induced Osteoarthritis by Inhibiting Nuclear Factor-κB via SIRT1
Source: Nutrients. 2022 Sep 24;14(19):3966. doi: 10.3390/nu14193966 (PMC9571821; doi:10.3390/nu14193966)

ADAMTS-4

MMP3

MMP13

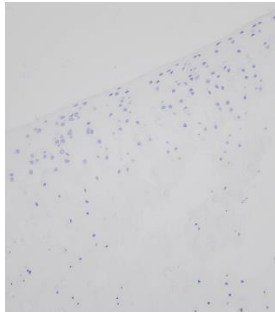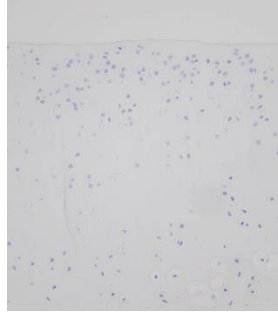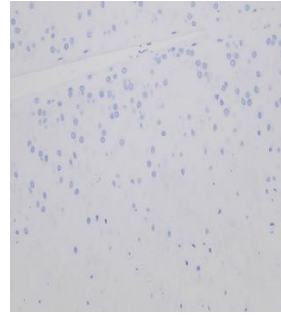

Type II collagen

Aggrecan

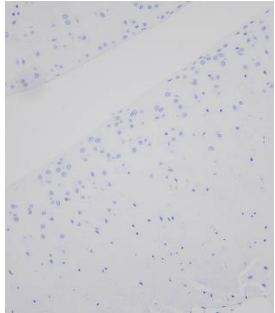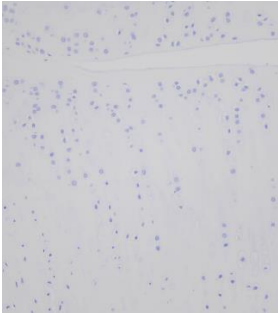

Figure S1: Negative control for immunohistochemistry

Supplement: Supplementary file 1 [file nutrients-14-03966-s001.zip › Figure S1.pdf]
